# The Roles of PPARs in the Fetal Origins of Metabolic Health and Disease

**DOI:** 10.1155/2008/459030

**Published:** 2008-01-15

**Authors:** William D. Rees, Christopher J. McNeil, Christopher A. Maloney

**Affiliations:** ^1^The Rowett Research Institute, Greenburn Road, Bucksburn, Aberdeen AB21 9SB, Scotland; ^2^Human Nutrition Unit, School of Molecular and Microbial Biosciences, The University of Sydney, NSW 2006, Australia

## Abstract

Beyond the short-term effects on fertility, there is increasing evidence that obesity or the consumption of an inappropriate diet by the mother during pregnancy adversely affects the long-term health of her offspring. PPAR and RXR isotypes are widely expressed in reproductive tissues and in the developing fetus. Through their interactions with fatty acids, they may mediate adaptive responses to the changes in the maternal diet. In the maturing follicle, PPAR-γ has an important role in the granulosa cells that surround the maturing oocyte. After fertilisation, PPAR-γ and PPAR-β/δ are essential regulators of placentation and the subsequent development of key metabolic tissues such as skeletal muscle and adipose cells. Activation of PPAR-γ and PPAR-β/δ during fetal development has the potential to modify the growth and development of these tissues. PPAR-α is expressed at low levels in the fetal liver, however, this expression may be important, as changes in the methylation of DNA in its promoter region are reported to take place during this period of development. This epigenetic modification then programmes subsequent expression. These findings suggest that two separate PPAR-dependent mechanisms may be involved in the fetal adaptations to the maternal diet, one, mediated by PPAR-γ and PPAR-β/δ, regulating cell growth and differentiation; and another adapting long-term lipid metabolism via epigenetic changes in PPAR-α to optimise postnatal survival.

## 1. INTRODUCTION

Human diets in the developed world have changed dramatically during the last century. An increase in the consumption of fat, coupled with a fall in physical activity, has led to unprecedented rates of obesity in Western populations. However, the complications associated with these changes in lifestyle extend beyond the present generation and threaten the next one. There is an overwhelming body of evidence showing that the diet and body composition of the mother modifies the risk of the offspring developing cardiovascular and metabolic diseases later in life [1]. Increased body weight and decreased physical activity are also associated with ovulatory dysfunction and reduced fertility [2, 3]. As the primary regulators of lipid metabolism at the cellular level, the peroxisome proliferator-activated receptor (PPAR) isotypes help to maintain metabolic homeostasis when the energy or lipid composition of the diet changes. The PPARs are widely expressed in the reproductive tissues and in the developing fetus, where by analogy with their function in adult tissues, they may mediate adaptations to the nutrient supply during reproduction. Recent studies of the mechanisms of metabolic programming have begun to shed light on the involvement of the PPARs in the fetal origins of health and disease [4–6]. In this review, we will consider the possible roles of PPAR isotypes and the related retinoid X receptor isotypes (RXR) in the developmental adaptations that occur in response to fluctuations in the maternal diet.

## 2. THE ROLE OF LIPID METABOLISM IN THE FETAL ORIGINS OF DISEASE

Much of the evidence from human and animal studies suggests that inappropriate energy metabolism during pregnancy has an adverse effect on fetal development and is an important factor in metabolic programming. In human populations, birth weight data is frequently used as a surrogate measure of fetal growth and hence the nutrient supply. Several studies have shown that there is a strong relationship between weight at birth and the risk of impaired glucose tolerance in adult life [7] and that there is a U-shaped relationship between birth weight and obesity in adult life [8]. Rapid catch-up growth in infancy following a period of fetal growth restriction carries the highest risk of central obesity in adulthood, particularly in babies that are thin at birth and small for gestational age. Importantly it is thinness at birth and not birth weight itself that explains the relationship between low birth weight and the long-term metabolic complications, suggesting that changes in the development of adiposity during fetal life is a critical factor [9]. At the other end of the spectrum, there is a positive association between birth weight and body mass index at age 20, suggesting that elevated birth weight is also associated with an increase in adiposity [10]. Mothers who are diabetic or develop serious gestational diabetes give birth to babies that are large for gestational age. These offspring of hyperglycaemic mothers have a much higher risk of developing metabolic syndrome in childhood, demonstrating a link between maternal blood glucose levels and perturbed metabolism in the offspring [11]. Thus, it appears that there are two different mechanisms underlying the development of glucose intolerance and obesity in adult life: one at the higher end of the birth weight spectrum, associated with maternal hyperglycemia, and another at the lower end associated with the development of adipose tissue [8].

Animal models for fetal programming also implicate lipid and carbohydrate metabolism in the programming process. Pertinent to this discussion of the role of PPARs in development are studies in which the maternal diet modifies lipid metabolism. Feeding rats a high-fat diet during gestation programmes glucose intolerance, pancreatic beta-cell dysfunction, and increases the body weight of their offspring [12, 13]. Other metabolic perturbations in gestation such as modest protein restriction, or iron deficiency also lead to persistent changes in the offspring. These also are linked indirectly to changes in lipid metabolism in the dam. In the case of protein restriction, triglyceride concentrations in the maternal plasma are increased in animals fed the low-protein rations and this is associated with changes in the expression of PPAR-*α* in the offspring [14]. This increase in plasma triglycerides can be modulated by the fatty acid composition of the diet [15], an intervention which also modifies the effects of protein deficiency on glucose tolerance in the offspring [16]. Micronutrients in the maternal diet are also important and there is evidence that their effects are also mediated indirectly through changes in lipid metabolism. For example, iron deficiency reduces triglyceride concentrations in the liver of the Fe-restricted fetuses by approximately 25% with corresponding changes in the expression of SREBP-1c and its downstream genes [17]. There are also reports that vitamin A deficiency during gestation is associated with impaired glucose tolerance in adult life [18].

**Figure 1:**
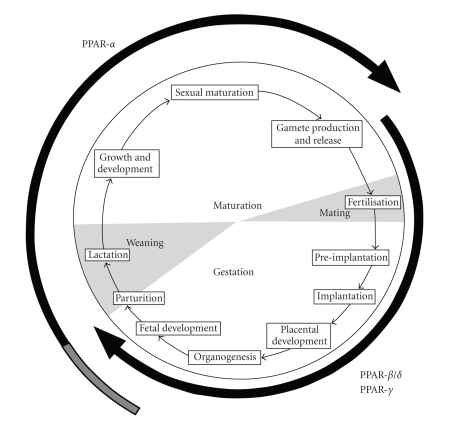
PPAR isotype expression and programming during the reproductive cycle. The PPAR-*β*/*δ* and PPAR-*γ* isotypes regulate the growth of key organs and manage the development of adipose tissue during fetal development. During the later stages of fetal development epigenetic programming of PPAR-*α* (represented by the grey section of the arrow) programmes long-term postnatal regulation of energy metabolism.

Both human and animal studies suggest that there are a number of critical windows in development where changes in the maternal diet can influence the long-term outcome of the offspring. These span the entire reproductive cycle from the preconception period when the germ cells mature right through gestation and into the lactation period (Figure 1).

## 3. PPARs DURING PRECONCEPTION DEVELOPMENT

Evolutionary forces favour animals able to regulate their fertility in response to the availability of nutrients in the environment. Metabolic status at the start of the reproductive cycle before conception is a good guide to subsequent success. Whilst these controls have developed to deal with famine, inappropriate responses to dietary excess or imbalance are more of concern in the modern world. Because of the links between body composition and infertility, there is considerable interest in the mechanisms by which nutrient sensors, such as the PPARs, regulate the maturation of the oocyte.

All of the PPAR isotypes are expressed in the rat ovary. PPAR-*γ* is found in the granulosa cells that surround and support the maturing oocyte. PPAR-*α* and PPAR-*β*/*δ* are present at lower levels in the thecal and stromal cells [19]. The low levels of the PPAR-*α* and PPAR-*β*/*δ* isotypes suggest that they play a role in basal ovarian function whereas the higher levels of the PPAR-*γ* isotype imply a more specific function in the granulosa cell [20]. However, PPAR-*γ* is not essential, as mice with a targeted deletion of the gene in granulosa cells are able to reproduce successfully, albeit with reduced fertility, related to a reduced implantation rate [21]. Instead PPAR-*γ* appears to be a negative regulator of follicular growth and differentiation. The viability of rat granulosa cells is reduced when they are treated with a specific PPAR-*γ* agonist, suggesting that PPAR-*γ* activation suppresses follicle development [22]. Recent studies also suggest that follicular functions are sensitive to dietary factors in vivo. Trans fatty acids increase the risk of ovulatory infertility when they replace the unsaturated fats that are commonly found in vegetable oils [23]. Since these fatty acids are able to activate PPAR-*γ*, the data suggest that it may be an important transducer.

Effects on ovulation, mediated by PPAR-*γ* in conjunction with RXR isotypes, may go beyond effects on fertility. Early embryonic development is dependent on stores of maternally derived factors passed from the granulosa cells to the oocyte during maturation. If these stores are depleted due to poor granulosa cell function, there may be an effect on the immediate postnatal development following fertilisation. A small change in growth during this early stage may be the start of a chain of events leading to long-lasting effects, such as elevated blood pressure in the offspring [24].

The PPARs are also expressed in the testis [20] where lipid metabolism and especially the *β*-oxidation of fatty acids are important for testicular function. Peroxisome proliferators, such as phthalates are known testicular toxicants. They interfere with the transcriptional activity of RAR-*α* in Sertoli cells by increasing the nuclear localisation of PPAR-*α* and increasing its transcriptional activity [25]. The extensive accumulation of neutral lipids in the testis has been observed in a number of mouse models in which key genes such as RXR-*β* have been deleted [26]. These finding suggest that the regulation of lipid metabolism by PPAR and RXR may be important in the regulation of male fertility. Unlike ovulation, the impact of high-fat diets and obesity on the function of PPARs during spermatogenesis is a relatively unexplored area. It is interesting to note that there has been a marked decrease in male fertility concomitant with the developing obesity epidemic suggesting that this is an area in need of further study.

## 4. PPARS DURING IMPLANTATION AND PLACENTATION

Following fertilisation there is a rapid differentiation of the early embryo into specialised cell types. This is the first stage of cellular differentiation when the tissues within the embryo begin to develop specialised metabolic functions. With this evolving complexity there is a requirement for mechanisms to maintain metabolic homeostasis between the different tissues. As the interface with the maternal circulation, the extraembryonic endoderm and then the placenta perform vital functions in regulating the nutrient supply to the developing tissues. The growth of the fetus is dependent on appropriate placental development, as a small placenta will restrict the availability of nutrients.

The PPAR isotypes play an important role in regulating the implantation of the embryo and the development of the placenta [27]. The mRNAs for RXR-*α*, RXR-*β* and PPAR-*γ* as well as the RXR-*β* and PPAR-*γ* proteins, have been detected in the trophectoderm and inner cell mass cells of intact and hatched blastocysts [28]. In mice, nutrients are transported through this extraembryonic endoderm prior to implantation. In cultures of trophoblast cells, activation of PPAR-*γ* or RXR with selective agonists enhances the uptake of free fatty acids and increases the accumulation of neutral lipids by increasing the expression of the FATP-4 transporter located in the brush-border membrane [29]. Thus, at this very early stage of development before the placenta is fully developed, the availability of substrates can modify the use of fatty acids by the embryo. At present, little is known about the impact of high-fat diets or obesity in this period and it remains to be seen if an increased utilisation of fatty acids at this stage has any long-term impact on the fetus.

The PPAR-*β*/*δ* and PPAR-*γ* isotypes also regulate fatty acid metabolism after the embryo has implanted and the placenta has developed. Fatty acids are used by the developing fetus for energy metabolism, membrane biosynthesis, and synthesis of signalling molecules. The PPAR-*β*/*δ* mRNA is ubiquitously expressed throughout the placenta including the labyrinth, the spongiotrophoblast, and the giant cells. Homozygous disruption of PPAR-*β*/*δ* results in the death of the majority of fetuses between days 9.5 and 10.5 of gestation. Pathological changes are mainly found in the giant cell layer of the placenta. The time of death corresponds to the period when PPAR-*β*/*δ* controls the differentiation and accumulation of lipid droplets in these cells [30]. In contrast, PPAR-*γ* is required for the development of the labyrinth layer of the placenta. The placentae of PPAR-*γ* null mice have impaired vascularisation [31] and fewer lipid droplets in the labyrinthine trophoblasts [32], resulting in embryonic lethality at about day 9.5 of gestation. Conversely, the activation of PPAR-*γ* by the administration of specific agonists in vivo reduces the thickness of the spongiotrophoblast layer, modifies the labyrinthine vasculature, and enhances fatty acid uptake and the expression of fatty acid transport proteins [33]. However, information on the action of nutritional factors is sparse. Metabolic perturbations such as those produced by experimental diabetes increase the expression of PPAR-*γ* and proteins that are regulated by it such as vascular endothelial growth factor [34]. These findings suggest that the PPAR-*γ* pathway might be involved in the impairment of placental development induced by high-glucose conditions. They also suggest that high-fat diets or obesity may also modify PPAR-*γ* signalling in the placenta due to high concentrations of lipids in the maternal circulation.

## 5. THE DEVELOPMENT OF ORGAN SYSTEMS

Further metabolic specialisation occurs within the fetus as the different organ systems develop. In the adult, the PPAR isotypes and isoforms play central roles in the metabolic interplay that occurs between the different organs. In the adult, adipose tissue, skeletal muscle, the liver, and pancreatic beta-cells are all involved in the regulation of glucose and lipid metabolism. The maternal diet has the potential to programme subsequent metabolism by modifying the development of these tissues during fetal development.

The association between thinness at birth and adult disease has been linked to the development of adipose tissue in utero, a process that involves both PPAR-*γ* and PPAR-*β*/*δ*. Animal studies suggest that the maternal diet does not influence either the proliferation or differentiation of preadipocyte cells in vitro [35]. Once preadipocytes have been isolated from the offspring, they proliferate and differentiate normally, suggesting that regulation must occur during fetal development. Many different transcription factors are involved in the commitment of mesenchymal stem cells to the adipocyte lineage [36]. Amongst these are PPAR-*β*/*δ*, which is expressed during the preadipose stages, and PPAR-*γ*, which is expressed as part of the mature adipocyte phenotype. Targeted deletions of the PPAR-*β*/*δ* and PPAR-*γ* genes in mice have demonstrated that both genes are essential for adipogenesis. The small numbers of PPAR-*β*/*δ* null mice that do not succumb to placental failure have an extremely lean phenotype, typified by a 2.5-fold reduction of abdominal fat mass compared with control littermates [37]. Similarly, PPAR-*γ* null mice, rescued by forming chimeras in which the placenta is formed from wild-type cells, die soon after birth because they are devoid of adipose tissue [32]. PPAR-*γ*-mediated signalling regulates adipogenesis in the adult by forming a positive feedback loop, sensitive to long-chain, saturated, and polyunsaturated fatty acids in the diet [38]. It is probable that this same system is able to regulate the development of fetal preadipose cells and adipocytes in situations where there are elevated levels of fatty acids supplied to the fetal tissues from either the maternal diet or through the mobilisation of maternal adipose reserves.

Altered muscle development may be an important element in prenatal programming of the metabolic syndrome. Skeletal muscles are a major site of carbohydrate and fatty acid metabolism and small changes induced during development have long-lasting effects. The offspring of rats fed high-energy diets (cafeteria diet) during gestation and lactation have fewer muscle fibres and more intramuscular fat, related to an increase in the expression of PPAR-*γ* mRNA in the muscle [39]. There is good evidence showing that both PPAR-*β*/*δ* and PPAR-*γ* regulate the expression of the genes involved myogenesis. Targeted expression of an activated form of PPAR-*β*/*δ* in the skeletal muscles of mice makes the animals resistant to obesity by increasing the numbers of oxidative muscle fibres [40], while the selective ablation of PPAR-*β*/*δ* induces obesity by reducing the oxidative capacity of the muscles [41]. In muscle cell cultures, PPAR-*β*/*δ* has been shown to regulate the expression of genes involved in fatty acid transport, beta-oxidation, and mitochondrial respiration [42]. Muscle specific ablation of the PPAR-*γ* gene in mice also produces animals that are obese and insulin resistant [43]. In contrast to the positive effects of PPAR-*β*/*δ* on myogeneisis, the overexpression of PPAR-*γ* in myoblast cultures has been shown to inhibit the formation of myotubes by suppressing the expression of muscle-specific myogenic proteins including myogenin, MyoD, and creatine kinase [44]. As a great deal of myogenesis takes place before birth, both PPAR-*β*/*δ* and PPAR-*γ* could be important regulators of fetal muscle development in response to lipids in the maternal diet.

Change in the size of the pancreatic islets due to an increase in beta-cells is an important feature of some animal models of fetal programming. PPAR-*γ* mediated signalling has been implicated in the regulation of beta-cell proliferation in adults. Mice in which the expression of the PPAR-*γ* gene was eliminated in beta-cells were found to have significant islet hyperplasia [45]. Paradoxically PPAR-*γ* agonists also enhance pancreatic growth [46] and the expression of key transcriptional activators required for beta-cell differentiation in cell cultures [47]. The reasons for these differences are unexplained. There is good evidence showing that changes in beta-cell expansion during the later stages of fetal development depend on glucocorticoids [48]. Thus, the role of PPAR-*γ* in the fetal pancreas remains unclear. However, the possibility remains that it may be important when the developing pancreas is exposed to high levels of fat from maternal obesity or high-fat diets.

The liver is the main site of PPAR-*α* expression in the adult, with much lower levels of the PPAR-*β*/*δ* and PPAR-*γ* isotypes found in this tissue. Homozygous disruption of the PPAR-*α*, PPAR-*β*/*δ*, and PPAR-*γ* genes has no effect on the development of the liver; and the offspring exhibit no apparent abnormalities [49]. However, PPAR-*α* is expressed in the fetal liver albeit at much lower levels than in the adult [50]; and as discussed below this fetal expression may be important in the programming of postnatal expression.

The RXR isotypes also plays a central role in organogenesis [51]. Recent studies of the mouse epidermis have suggested that 9-cis retinoic acid is not the in vivo ligand of RXR [52]. The actions of various pharmacological agents and the observation that keratinocytes do not contain retinoids suggest that fatty acids are the natural RXR ligand and that RXR is acting as a lipid sensor. Thus, it is possible that the same fatty acids are able to activate both partners of a PPAR:RXR heterodimer. If these findings hold for PPAR:RXR heterodimers in other tissues then this represents a clear mechanism by which the availability of fatty acids can influence fetal development.

## 6. THE PROGRAMMING OF PPAR-*α* EXPRESSION

Persistent alterations to the phenotype of the offspring imply stable changes in gene expression. Candidate genes for such effects arise from studies showing altered gene expression in the offspring of laboratory animals fed restricted diets. There is accumulating evidence that there are long-term changes in the stable expression of PPAR-*α* [14] and of genes regulated by it, including acetyl-CoA carboxylase and fatty acid synthase [16, 53]. A change in the expression of these genes is associated with impaired lipid homeostasis in the adult. Recent studies have found evidence for epigenetic changes in the PPAR-*α* gene which may account for this programming [4]. Analysis of genomic DNA using methylation specific restriction enzymes suggests that the methylation of the exon 1 promoter was approximately 20% lower in the offspring of rats fed a low-protein diet in gestation. At the same time, there was a 10-fold increase in the mRNA for PPAR-*α*. These changes were specific for PPAR-*α* as there was no change in the methylation status of the PPAR-*γ* gene. Similar epigenetic changes induced during fetal development and persisting into adult life with long-lasting effects on the physiological mechanisms have been demonstrated with the glucocorticoid receptor [54].

Nutrient sensitive transcriptional activators, such as the PPAR-*α*, are able to determine local chromatin structure through interactions with coactivator proteins. Indeed, these interactions are an essential component of the mechanism of transcriptional activation [55]. Even when there is no ligand present, PPARs form heterodimers with RXR*α* which bind to DNA in association with a number of corepressor proteins. Binding of a ligand to a PPAR dissociates the corepressor protein complex, releasing the PPAR:RXR heterodimer which then sequentially associates with various transcriptional coactivator proteins. This protein complex modifies histone and chromatin structure, making the DNA accessible for transcription while at the same time recruiting RNA polymerase II and activating the transcriptional machinery. The proteins involved in the coactivator complex include PGC-1 histone acetyl transferases, histone deacetylases and methyl transferases [55]. At present, there are no reports of coactivators with transcriptional functions specific to the PPAR subfamily. Individual coactivators are shared by many transcription factors and are involved in numerous signalling pathways [56, 57]. For example, the nuclear receptor coactivator PBP (PPAR-binding protein) functions as a coactivator for other members of the nuclear receptor family. A targeted deletion of the PBP gene in hepatocytes reduces the association of other unrelated cofactors, especially the cyclic-AMP responsive element binding protein and thyroid hormone receptor-associated proteins to the PPAR-*α* dependent mouse enoyl-CoA hydratase/L-3-hydroxyacyl-CoA dehydrogenase gene promoter [58]. Within the nuclear receptor coactivation complex there are some proteins, which do not directly bind to nuclear receptors but are present in the complex due to their binding to other coactivators. Amongst these are proteins that can methylate histones. It has also been suggested that changes in the recruitment of the Dnmt-1 methyl transferase to the promoter during development may be responsible for the modification of DNA methylation at the glucocorticoid receptor [59].

Thus, interactions between PPAR-*α* and its ligands in the liver during fetal development may be important in adapting chromatin structure, and hence long-term expression, to the nutrient supply likely to be encountered by the fetus in postnatal life. Because these modifications occur before PPAR-*α* is required for metabolic regulation, this may be a molecular mechanism which establishes the sensitivity of the developing tissue to nutrient signals. These modifications to the metabolic phenotype may be beneficial when nutrients are limited, as it provides a mechanism that will adapt the response of the offspring to a poor diet in the postnatal environment. Equally, when the diet is high in fat and carbohydrates, hepatic metabolism will be well adapted to direct excess fat towards storage in adipose tissue and prevent some of the adverse effects of lipotoxicity.

## 7. CONCLUDING REMARKS

PPAR and RXR isotypes have an essential role in the homeostatic mechanisms that maintain energy metabolism in the adult. There is now increasing evidence that they ensure that the metabolic tissues of the fetus develop in a controlled way during gestation. It appears that there may be two different PPAR-mediated mechanisms involved in the fetal origins of health and disease. One is mediated via PPAR-*γ*, which regulates the growth of key organs and manages the development of adipose tissue during fetal development. The other is mediated via PPAR-*α* in which epigenetic control preprogrammes long-term regulation of energy metabolism.

Bioactive factors such as lipids, carbohydrates, amino acids, as well as lipid-derived hormones crossing the placental barrier may disrupt this careful balance in metabolism. Critically, regulatory systems that have evolved to deal with famine are poorly suited to deal with nutrient excess. High levels of lipid, either from the diet or derived from excessive maternal stores may overwhelm the protective mechanisms offered by the PPAR receptors. Once inappropriate control points are established, then metabolic balance will be disturbed for the remainder of life. Insulin resistance programmed at fetal stages will become more pronounced with age, ultimately leading to the development of metabolic disease.
